# Exposure to Essential and Toxic Elements via Consumption of *Agaricaceae*, *Amanitaceae*, *Boletaceae,* and *Russulaceae* Mushrooms from Southern Spain and Northern Morocco

**DOI:** 10.3390/jof8050545

**Published:** 2022-05-23

**Authors:** Marta Barea-Sepúlveda, Estrella Espada-Bellido, Marta Ferreiro-González, Hassan Bouziane, José Gerardo López-Castillo, Miguel Palma, Gerardo F. Barbero

**Affiliations:** 1 Department of Analytical Chemistry, Faculty of Sciences, University of Cadiz, Agrifood Campus of International Excellence (ceiA3), IVAGRO, 11510 Puerto Real, CA, Spain; marta.barea@gm.uca.es (M.B.-S.); marta.ferreiro@uca.es (M.F.-G.); miguel.palma@uca.es (M.P.); gerardo.fernandez@uca.es (G.F.B.); 2 Laboratory of Applied Botany, Department of Biology, Faculty of Sciences, University Abdelmalek Essaâdi, Mhannech II, Tetouan 2121, Morocco; hasbouz@hotmail.com; 3 Unidad de Protección de la Salud, Distrito Sanitario Granada-Metropolitano, Consejería de Salud y Familias, Junta de Andalucía, 18150 Gójar, GR, Spain; joseg.lopez.sspa@juntadeandalucia.es

**Keywords:** wild edible mushrooms, metallic elements, metalloids, risk assessment, human health, organic food

## Abstract

The demand and interest in mushrooms, both cultivated and wild, has increased among consumers in recent years due to a better understanding of the benefits of this food. However, the ability of wild edible mushrooms to accumulate essential and toxic elements is well documented. In this study, a total of eight metallic elements and metalloids (chromium (Cr), arsenic (As), cadmium (Cd), mercury (Hg), lead (Pb), copper (Cu), zinc (Zn), and selenium (Se)) were determined by ICP-MS in five wild edible mushroom species (*Agaricus silvicola*, *Amanita caesarea*, *Boletus aereus*, *Boletus edulis,* and *Russula cyanoxantha*) collected in southern Spain and northern Morocco. Overall, Zn was found to be the predominant element among the studied species, followed by Cu and Se. The multivariate analysis suggested that considerable differences exist in the uptake of the essential and toxic elements determined, linked to species-intrinsic factors. Furthermore, the highest Estimated Daily Intake of Metals (EDIM) values obtained were observed for Zn. The Health Risk Index (HRI) assessment for all the mushroom species studied showed a Hg-related cause of concern due to the frequent consumption of around 300 g of fresh mushrooms per day during the mushrooming season.

## 1. Introduction

Mushrooms are the fruiting bodies or sporocarps of a group of eukaryotic organisms classified in the division *Basidiomycota* and *Ascomycota* of the Kingdom Fungi [1,2,3,4]. Although mushrooms have traditionally been considered a delicacy in gastronomy for their palatability, advances in the scientific field have led to a new nutritional approach to their culinary use, as their properties suggest that mushroom consumption may influence the control and modulation of several functions of the organism when introduced in the diet [5,6]. Therefore, the demand and interest in mushrooms have increased among consumers in recent years due to a better understanding of their benefits and a greater awareness of the need to include more nutritious options for healthier habits. Likewise, since edible mushrooms play an important role as a source of income for many companies and local communities, the increase in its demand has also contributed to the economic growth of this market and, consequently, to that of the countries [2]. In 2020, the global mushroom market size was valued at $46.1 Bn, and it is estimated to expand at a compound annual growth rate (CAGR) of 9.5% from 2021 to 2028 [7]. In turn, in Spain, the 2020 annual household consumption panel data issued by the Ministry of Agriculture, Fisheries, and Food indicated that the quantities consumed nationwide totaled 70,221 tons [8]. Among the mushrooms marketed and consumed, edible wild mushrooms currently attract a higher level of attention, as most of them cannot be cultivated due to their complex and specific symbiotic lifestyle [3,9,10]. For this reason, freshly wild-growing mushrooms are high-valued seasonal foods, widely consumed by many people who enjoy life outside the urban areas [11,12]. Worldwide, the most consumed and appreciated wild mushroom species belong to the families *Russulaceae* (e.g., *Russula cyanoxantha* and *Lactarius deliciosus*), *Boletaceae* (e.g., *Boletus edulis* and *Boletus aereus*), *Amanitaceae* (e.g., *Amanita caesarea*), *Cantharellaceae* (e.g., *Cantharellus cibarius*), and *Agaricaceae* (e.g., *Agaricus silivicola* and *Macrolepiota procera*). 

Unlike the cultivated ones, wild-growing mushrooms fructify and mature exposed to ambient conditions. Anthropogenic activities such as pesticide use, mining, and fossil fuel combustion produce large quantities of pollutants that affect environmental homeostasis when they enter the air, water, or soil [13,14]. Of all the pollutants found in the environment, metallic elements and metalloids are considered to represent one of the most dangerous for public health due to their high persistence and tendency to bioaccumulate in the trophic chain. Metallic elements and metalloids such as mercury (Hg), lead (Pb), cadmium (Cd), chromium (Cr), and arsenic (As) have no biological functions and are therefore considered non-essential and harmful to the body even at low concentrations. For example, sublethal Pb poisoning causes neurological defects and renal dysfunction [15,16]. Nonetheless, metallic elements and metalloids such as copper (Cu), zinc (Zn), and selenium (Se) are known to be essential nutrients for different physiological functions in the body, so the lack of any of them could have repercussions on biological processes, compromising human health. For example, symptoms of Zn deficiency include growth retardation, hair loss, diarrhea, eye and skin lesions, loss of appetite, and delayed wound healing [17]. The ability of wild mushrooms to accumulate metallic elements and metalloids in their tissues is well documented by other authors [18,19,20,21,22,23,24,25,26,27], showing that the concentrations of these contaminants in the wild-growing species are considerably greater than those in the soil where they grew [28]. The mechanism that enables mushrooms to accumulate metallic elements and metalloids could be explained via the network of hyphae located in the upper soil horizon [29,30]. The hyphae, consisting of elongated tubular cells enveloped by a chitin wall, are widely spread over the bioavailable area and, under specific conditions, accumulate metal ions [31]. In addition, this process seems to be influenced by environmental factors that influence the bioavailability of these pollutants in the environment (pH, organic matter, texture, and metal concentration in the soil) and intrinsic factors (taxon, morphology, size, fruiting body part, stage of development, and mycelial age) [32]. Thus, owing to the important role that mushrooms are acquiring in diets, the presence of high concentrations of metallic elements and metalloids in this food constitutes a matter of great relevance within the scientific community.

Based on this background, the present study aimed to determine the composition of five toxic elements (Cr, As, Cd, Hg, and Pb) and three essential elements (Cu, Zn, and Se) in five high-valued species of wild-growing edible mushrooms (*A. silvicola*, *A. caesarea*, *B. aereus*, *B. edulis,* and *R. cyanoxantha*) collected in southern Spain and northern Morocco. The latter was selected as a sampling location due to its proximity to southern Spain and the increased mycological activity during the mushrooming season in some locations in its northern region. Unsupervised multivariate techniques, such as Hierarchical Cluster Analysis (HCA) and Principal Component Analysis (PCA), were applied to study the influence of the source area and species-intrinsic factors over the metallic elements and metalloids. Furthermore, consumer exposure and health implications were assessed using two well-known safety criteria: the Estimated Daily Intake of Metals (EDIM) and the Health Risk Index (HRI).

## 2. Materials and Methods

### 2.1. Mushroom Sampling

Edible mushroom fruiting bodies were collected in 2017 and 2018 during the mushrooming season from different locations in southern Spain and northern Morocco. All sampled locations corresponded to natural forests, and therefore, the edible mushroom samples were all wild. A total of 16 samples of 5 mushroom species were collected and analyzed. Specimens studied were authenticated based on their morphological distinctive characteristics, which are unique and unmistakable from other mushroom species and families. Sample collection and preparation were carried out following the process previously described in earlier work conducted by our research group [18,33]. At least 10 complete specimens were collected *in situ* in each of the areas to form a representative set of samples from each geographic location studied. Upon arrival at the laboratory, the samples were adequately prepared for their analysis. To this end, all the fruiting bodies collected were washed with deionized water and dried in an oven at 50 °C for 48 h until a constant weight was reached. Finally, the samples were homogenized using an agate mortar and stored in clean polyethylene (PE) bottles, perfectly labeled according to species and sampling area. The habitat, year of collection, sample location, and families of the mushrooms studied are shown in Table 1. 

### 2.2. Chemicals and Solvents

The reagents used for the acid digestions of the samples were of high analytical grade and acquired from SCP Science (Montreal, Quebec, Canada): HCl PlasmaPURE (34— 37%), HNO_3_ PlasmaPURE (67—69%), and Sigma-Aldrich (St. Louis, MO, USA): H_2_O_2_ (≥ 30%). The solutions were prepared using nanopure water obtained by passing twice-distilled water through a Milli-Q system (18 MΩ/cm, Millipore, Bedford, MA, USA).

### 2.3. Acid Digestion Procedure

Acid digestions were performed in a DigiPREP Jr block digestion system from SCP Science (Montreal, Quebec, Canada) equipped with a 24-position graphite heating block for 50 mL polypropylene (PE) digestion tubes (DigiTubes; SCP Science; Montreal, Quebec, Canada). The digestion procedure employed here was based on the conditions established in previous investigations by our research group [33]. The dried and powdered subsamples (0.25 g) were first placed in the digestion tubes with 5 mL of HNO_3_, 2 mL of HCl, and 2 mL of nanopure water. Next, they were digested by applying a stepwise temperature increase procedure for 20 min up to 65 °C and maintaining this temperature for a total of 30 min. The second digestion was performed after a cooling step by adding 3 mL of H_2_O_2_, gradually increasing the temperature for 30 min up to 110 °C, and holding it for a total of 60 min. Prior to analysis, the digested samples were filtered through a 0.45-μm filter (DigiFILTER; SCP Science; Montreal, Quebec, Canada) using a –600 mbar vacuum port. They were then transferred to a clean 50-mL volumetric DigiTube, which was completed with nanopure water to 50 mL. All samples were carried out in triplicate.

### 2.4. Analysis

The concentrations of Cr, As, Cd, Hg, Pb, Cu, Zn, and Se in the mushroom samples were determined with an Inductively Coupled Plasma-Mass Spectrometer (Thermo X Series II ICP-MS, Waltham, MA, USA) equipped with a concentric nebulizer, cyclonic spray chamber, quadrupole mass analyzer, and collision/reaction cell. The Xt interface, kinetic energy discrimination (KED), and H_2_ (7%)/He CCT were applied throughout the analyses. The instrumental conditions of the ICP-MS are shown in Table 2. 

An internal standard of ^103^Rh, ^72^Ge, ^191^Ir, ^209^Bi, and ^45^Sc prepared from individual solutions (SCP Science; Montreal, Quebec, Canada) of 1000 μg mL^−^^1^ was used to correct temporal variations in signal intensity during the analyses. Moreover, the analytical methodology used to determine the metallic elements and metalloids concentrations were validated through triplicates, blanks, and a certified reference material (CRM), the *Boletus edulis* CS-M-3 powder control material (Institute of Nuclear Chemistry and Technology; Warsaw, Poland). No interferences were found relevant for the quantified elements in blanks. The recovery levels in the reference material (CS-M-3) were in an acceptable range of 70–130%. Limits of detection (LOD) for Cr, As, Cd, Hg, Pb, Cu, Zn, and Se were within 0.002–0.1 mg kg^−^^1^ dry weight (DW).

### 2.5. Estimated Daily Intake of Metals 

For each sample analyzed, the Estimated Daily Intake of Metals (*EDIM*) was calculated using the concentration data expressed in mg kg^−^^1^ dry weight (DW), as shown in the following Equation (1):

(1) $$EDIM=\frac{C_{metal}\cdot D_{food\;intake}}{BW}$$

where *C*_*metal*_ is the metallic element or metalloid concentration (mg kg^−^^1^) in the fruiting body, *D*_*food intake*_ refers to the mushroom daily intake, and BW is the average person’s body weight in kg. Following Liu et al. (2015) and Sarikurkcu et al. (2020) [2,14], we considered a portion of 300 g of fresh mushrooms (30 g of dried mushrooms per day) and a regular consumer of 70 kg body weight.

### 2.6. Health Risk Index 

The Health Risk Index (HRI) was calculated by following Equation (2) to assess the potential risk to human health due to exposure to the metallic elements and metalloids in the different species of edible wild-growing mushrooms studied:

(2) $$\mathrm{HRI}=\frac{EDIM}{R_{f}D}$$

where *EDIM* is the daily intake of metals via consumption of the studied mushrooms, and *R_f_D* is the maximum acceptable daily oral dose of a toxic substance. According to the data provided by Sarikurkcu et al. (2020) and the U.S. EPA Integrated Risk Information System (IRIS) data, the Reference Doses (*R_f_D*) for Cr, As, Cd, Hg, Pb, Cu, Zn, and Se are 3, 0.3, 1, 0.3, 3.5, 40, 300, and 0.5 μg kg^−^^1^ body weight per day, respectively.

### 2.7. Software and Multivariate Analysis

An unsupervised multivariate analysis was applied to identify clustering trends among the species studied. To this end, the average concentrations in mg kg^−1^ of the elements determined were used as variables, obtaining a data matrix, D*_nxm_*, where *n* is the number of samples (*n* = 16), and *m* is the number of metallic and metalloid elements determined (*m* = 8). The data matrix was normalized using Min–Max normalization. A hierarchical cluster analysis (HCA) and Principal Components Analysis (PCA) were performed first. HCA allows the hierarchical classification of the data according to their similarity. The Euclidean distance was selected for the inter-individual similarity matrix calculation and Ward’s method as the inter-cluster measure. In this study, the choice of Ward’s method was settled by comparing the agglomerative coefficient obtained with different linkage methods (Average, Complete, Unique, and Ward). This coefficient enables us to find the linkage method that identifies the strongest clustering structure. An agglomerative coefficient equal to 1 is the highest value, indicating a strong clustering structure. In this case, Ward’s method was the linkage method that presented an agglomerative coefficient closest to 1, being 0.76. PCA is an unsupervised multivariate technique that allows for reducing the dimensionality of the data, so it is used together with HCA as an exploratory technique.

The multivariate analysis was performed with Rstudio (R version 4.0.5, Boston, MA, USA). The hierarchical Cluster Analysis (HCA) was carried out using the *hclust* function from the *stast* package. The selection of the Linkage method for the HCA was established by using the *agnes* function of the *clust* package. The HCA results were plotted in a graphical combination of the resulting sample and variable dendrograms with a heatmap using the *heatmap.2* function of the *gplots* package. The Principal Components Analysis (PCA) was performed using the *prcomp* function from the *stats* package. The *fviz_eig* function from the *factoextra* package was used to extract and visualize the output of this multivariate data analysis. The scores and loadings obtained from the PCA were graphically displayed using the *ggplot* function from the *ggplot2* package. Furthermore, the *ggplot* function from the *ggplot2* was also applied for the bar chart representation of the Health Risk Index (HRI) results.

## 3. Results and Discussion

### 3.1. Metallic Elements and Metalloids Content in Mushrooms

The metallic and metalloid concentrations of the wild edible mushrooms studied are given in Table 3, expressed as the mean of triplicates in mg kg^−1^ dry weight (DW). All the elements included in our study were detected in all the mushroom samples, except for As, which was below the Limit of Detection (LOD) in samples #15 and #16, which were both *Boletus aereus* from Parc Naturel of Bouhachem (Chaouen Morocco). In general, it was observed that Zn was the predominant element in most of the cases, followed by Cu and Se. The other metallic elements and metalloids were detected in relatively lower concentrations.

#### 3.1.1. Essential Metallic Elements and Metalloids

In the present study, the highest Cu concentrations (Table 3) were observed in sample #9 *A. silvicola* (147 ± 2.07 mg kg^−1^ DW) collected in Cortes de la Frontera (Malaga, Spain), while the lowest levels determined for this metallic element were found in sample #11 *B. edulis* (18.6 ± 0.257 mg kg^−1^ DW) collected in Puerto de Galiz (Cadiz, Spain). The Cu results obtained in this study were compared with those reported by other authors. For *R. cyanoxantha*, A. R. Zsigmond et al. (2020) indicated concentrations of 37.1– 55 mg kg^−1^ DW for samples collected in Romania [34]. Sarikurkcu et al. (2010) established Cu levels of 16.4 mg kg^−1^ DW in A. *caeasera* mushrooms from Turkey [35]. On the other hand, A. Demirbaş (2001) reported concentrations of 6.24 mg kg^−1^ DW for A. *silvicola* samples collected in Turkey [29]. M. G. Alaimo et al. (2018) indicated Cu concentrations of 31 mg kg^−1^ DW (cap) and 16 mg kg^−1^ DW (steam) in *B. aereus* mushrooms from Italy [36]. Meanwhile, J. Falandysz et al. (2008) reported concentrations in a range of 26–57 mg kg^−1^ DW (caps) for *B. edulis* samples collected in Poland [37]. The Cu results obtained in the present research were in general above the data observed in the literature.

The highest/lowest Zn concentrations (Table 3) determined were observed in samples #8 *A. silvicola* (213 ± 3.62 mg kg^−1^ DW) from Parc Naturel Bouhachem (Chaouen, Morocco) and #2 *R. cyanoxantha* (51.2 ± 0.873 mg kg^−1^ DW) from Cortes de la Frontera (Cadiz, Spain), respectively. The Zn results obtained in this research were compared with those reported by previous studies. A. R. Zsigmond et al. (2020) indicated concentrations in the range of 74.4–108 mg kg^−1^ DW for *R. cyanoxantha* samples collected from geographical locations in Romania [34]. For *A. caesarea*, Sarikurkcu et al. (2010) reported 123.8 mg kg^−1^ DW concentrations for samples collected in Turkey [35]. Meanwhile, A. Demirbaş (2001) established Zn concentrations of 25.6 mg kg^−1^ DW for *A. silvicola* mushrooms from Turkey [29]. On the other hand, M. G. Alaimo et al. (2018) indicated concentrations of 125 mg kg^−1^ DW (cap) and 31 mg kg^−1^ DW (steam) in *B. aereus* mushrooms from Italy [36]. J. Falandysz et al. (2008) reported Zn concentrations in a range of 150–210 mg kg^−1^ DW (caps) for *B. edulis* samples collected in Poland [37]. The results obtained for Zn through our analysis were in most of the cases above those reported by other researchers. 

Regarding Se (Table 3), the highest concentrations were obtained in sample #14 *B. aereus* (76.8 ± 1.46 mg kg^−1^ DW) from Puerto de Galiz (Cadiz, Spain) and the lowest in sample #13 *B. aereus* (0.278 ± 0.003 mg kg^−1^ DW) from Valdeinfierno (Cadiz, Spain). The Se concentrations observed in this study were compared with those indicated by other authors in previous research. For *R. cyanoxantha*, L. Cocchi et al. (2006) reported concentrations of 2.18 mg kg^−1^ DW in samples of this species collected in Italy [38]. Likewise, L. Cocchi et al. (2006) indicated concentrations of 3.30 mg kg^−1^ DW in *A. caesarea* samples from Italy [38]. On the other hand, M. Tuzen et al. (2007) established Se concentrations of 1.23 mg kg^−1^ DW for *A. silvicola* mushrooms collected in Turkey [39]. Moreover, L. Cocchi et al. (2006) reported concentrations of 24.6 and 30.8 mg kg^−1^ DW for *B. aereus* and *B. edulis* samples, collected in Italy [38]. Comparing the results obtained with those previously reported by these authors, it has been observed that the Se concentrations here were consistent with the literature.

#### 3.1.2. Toxic Metallic Elements and Metalloids

Among the species studied, the highest Cr concentrations (Table 3) were determined in sample #3 *R. cyanoxantha* (10.2 ± 0.079 mg kg^−1^ DW) collected in Cortes de la Frontera (Malaga, Spain), whereas the lowest were in sample #10 *A. silvicola* (0.531 ± 0.051 mg kg^−1^ DW) from Cortes de la Frontera (Malaga, Spain)^2^. The Cr results obtained in this study were compared with those reported by other authors. For *R. cyanoxantha*, A. R. Zsigmond et al. (2020) indicated concentrations of 0.40 mg kg^−1^ DW for samples collected in Romania [34]. M. Yamaç et al. (2007) reported Cr levels of 0.54 mg kg^−1^ DW in the *A. caesarea* mushroom from Turkey [40]. Meanwhile, M. A. García et al. (2013) established concentrations of 3.3 mg kg^−1^ DW in *A. silvicola* samples from northern Spain [41]. Moreover, M. A. García et al. (2013) indicated concentrations of 3.8 mg kg^−1^ DW for both *B. aereus* and *B. edulis* mushrooms collected in northern Spain regions [41]. The Cr levels found in the samples studied were by the concentrations reported in the literature.

Regarding As (Table 3), the highest concentration was registered in sample #9 *A. silvicola* (3.78 ± 0.005 mg kg^−1^ DW) from Cortes de la Frontera (Malaga, Spain)^1^ and the lowest levels in sample #3 *R. cyanoxantha* (0.111 ± 0.003 mg kg^−1^ DW) collected in Cortes de la Frontera (Malaga, Spain). The As results obtained in this study were compared with those reported by other authors. L. Cocchi et al. (2006) indicated concentrations of 0.10 mg kg^−1^ DW for *R. cyanoxantha* samples collected from geographical locations in Italy [38]. For *A. caesarea*, G. M. Chiocchetti et al. (2020) established As concentrations in a range of 0.275 — 0.706 mg kg^−1^ DW for samples from Spain [42]. A. Demirbaş (2001) reported concentrations of 0.76 mg kg^−1^ DW in *A. silvicola* mushrooms collected in Turkey [29]. Moreover, L. Cocchi et al. (2006) indicated concentrations of 0.39 and 0.10 mg kg^−1^ DW for *B.s aereus* and *B. edulis* samples, collected in Italy [38]. Thus, the As results obtained through our analysis agree with those reported in previous studies.

Concerning Cd (Table 3), the highest/lowest concentrations were determined in samples #9 A. silvicola (25.9 ± 0.418 mg kg^−1^ DW) collected from Cortes de la Frontera (Malaga, Spain)^1^ and #2 *R. cyanoxantha* (0.223 ± 0.030 mg kg^−1^ DW) from Sendero El Palancar (Cadiz, Spain), respectively. The Cd results obtained in this research were compared with those reported by previous studies. M. J. Melgar et al. (2016) indicated concentrations of 0.28 mg kg^−1^ DW for *R. cyanoxantha* samples collected in northern Spain regions [27]. On the other hand, Sarikurkcu et al. (2010) reported Cd concentrations of 0.54 mg kg^−1^ DW in A. caesaera samples collected in Turkey [35]. For A. silvicola, A. Demirbaş (2001) indicated Cd levels of 1.04 mg kg^−1^ DW in samples from Turkey [29]. Furthermore, M. J. Melgar et al. (2016) established concentrations of 0.93 and 0.64 mg kg^−1^ DW for *B. aereus* and *B. edulis* samples, respectively, collected in northern Spain regions [27]. The Cd results obtained in the present research were generally above the data observed in the literature.

The maximum Hg concentrations (Table 3) in this study were observed in sample #15 *B. aereus* (11.1 ± 0.489 mg kg^−1^ DW) collected in Parc Naturel Bouhachem (Chaouen, Morocco) and the minimum in sample #8 A. silvicola (1.19 ± 0.023 mg kg^−1^ DW) from Parc Naturel Bohuachem (Chaouen, Morocco). The Hg concentrations observed in this study were compared with those indicated by other authors in previous research. For *R. cyanoxantha*, L. Cocchi et al. (2006) reported concentrations of 1.31 mg kg^−1^ DW in samples of this species from Italy [38]. Ostos et al. (2016) reported Hg concentrations of 0.81 mg kg^−1^ DW (stem) and 2.03 mg kg^−1^ DW (caps) in *A. caesarea* samples collected in southern Spain [24]. A. Demirbaş (2001) reported concentrations of 0.15 mg kg^−1^ A. silvicola mushrooms from Turkey [29]. On the other hand, M. J. Melgar et al. (2009) established concentrations of 3.0 and 2.0 mg kg^−1^ DW for *B. aereus* and *B. edulis* samples, collected in northern Spain [43]. Therefore, the Hg content levels determined in our study for these species were found to be above the data presented by the mentioned studies. 

The highest Pb concentrations (Table 3) were found in sample #9 A. silvicola (1.21 ± 0.019 mg kg^−1^ DW) collected from Cortes de la Frontera (Malaga, Spain) and the lowest in sample #15 *B. aereus* (0.047 ± 0.003 mg kg^−1^ DW) from Parc Naturel Bouhachem (Chaouen, Morocco). The Pb results obtained in this research were compared with those reported by previous studies. A. Demirbaş (2001) indicated concentrations in the range of 2.05 mg kg^−1^ DW for *R. cyanoxantha* samples collected from geographical locations in Turkey [29]. For *A. caesaera*, Sarikurkcu et al. (2010) reported concentrations of 5.0 mg kg^−1^ DW in samples from Turkey [35]. A. Demirbaş (2001) reported concentrations of 0.92 mg kg^−1^ DW in A. silvicola samples collected in Turkey [29]. On the other hand, M. A. García et al. (2009) established concentrations of 0.70 and 0.67 mg kg^−1^ DW for *B. aereus* and *B. edulis* samples collected in northern Spain [44]. We observed that our Pb concentrations for these species were mostly lower than those reported in the literature. 

### 3.2. Multivariate Analysis

**Figure 1:**
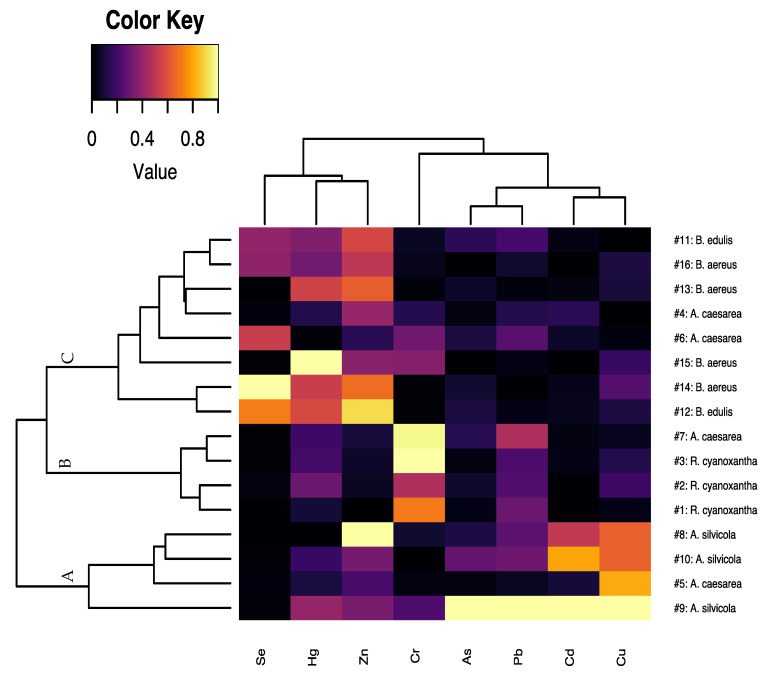
Graphical combination of the resulting HCA dendrograms with a heatmap to identify clustering trend patterns among the studied mushroom species based on the content of the eight elements determined.

The results obtained through HCA using the Wards method and the Euclidean distance are shown in Figure 1 using a graphic representation based on the combination of the resulting dendrograms of variables and samples, together with a heatmap. As can be seen in Figure 1, a color scale ranging from purple to yellow was established to visualize more intuitively the levels of Cr, As, Cd, Hg, Pb, Cu, Zn, and Se in the mushroom samples. Thus, darker shades of purple would indicate a low concentration of the given element, and gradual changes to yellow would indicate an increase in concentration. As can be seen, three main clusters (A, B, and C) were obtained. Based on the information provided by the heatmap, these three clusters are differentiated from each other according to the Cr, As, Cd, Hg, Pb, Cu, Zn, and Se content. Specifically, clusters A and B are separated from cluster C due to a lower content of Se, Hg, and Zn and a higher content of Cd and Cu in the mushroom samples grouped therein. On the other hand, cluster A differs from cluster B mainly because the samples included in it have a lower Cr content and a higher Cd and Cu content. From the results obtained in the HCA, it was observed that there was a strong tendency of metal and metalloid accumulation related to the mushroom species and family, regardless of the geographical location where they were collected. In this sense, cluster A is formed by all the *A. silvicola* samples studied, while cluster B is constituted by all the *R. cyanoxantha* samples analyzed. Cluster C is formed by both *B. aereus* and *B. edulis* samples, which are both species belonging to the *Boletaceae* family. Thus, it is possible to observe that although environmental factors affect the bioavailability of metallic elements and metalloids in the medium, intrinsic factors such as species and family have a greater influence on the phenomenon of metal accumulation in mushrooms. However, this clustering is not completely consistent, since the *A. caesarea* samples were grouped among the three main clusters and not in an exclusive cluster like the rest of the species studied.

**Figure 2:**
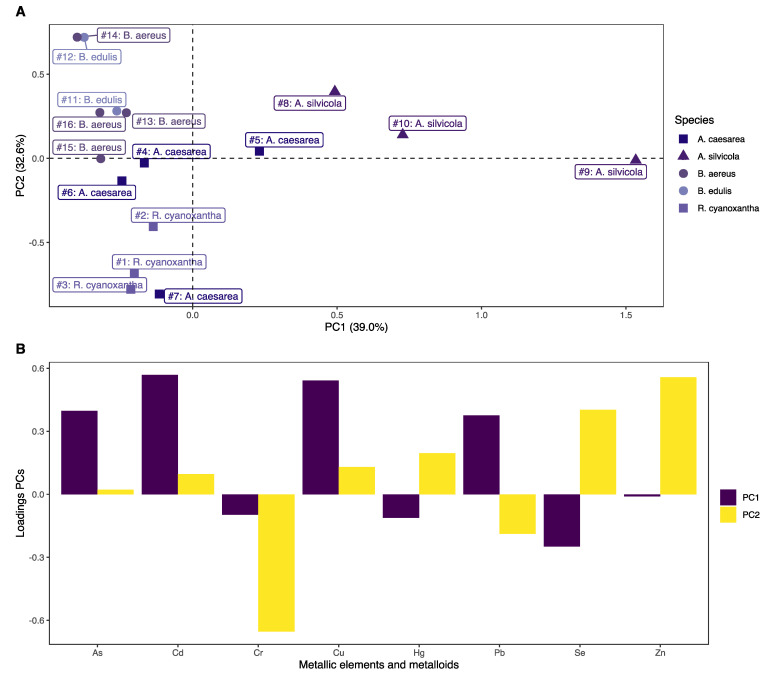
(**A**) Score obtained for PC1 and PC2 for all the samples (n = 16); (**B**) Loadings obtained in PC1 and PC2.

A Principal Component Analysis (PCA) was also carried out to corroborate this clustering trend. Figure 2A shows the plot of the scores obtained for the first two principal components (PC1 and PC2) for all samples (n = 16), and Figure 2B shows the plot of the loadings obtained for each principal component (PCs). Principal components 1 and 2 explained 39.0% and 32.6% of the variance of the data, respectively, which implies a total accumulated variance of 71.6% between both. It can be observed that PC1 (Figure 2A) allowed the separation of the *A. silivicola* samples from the rest of the species studied. For their part, Cd and Cu were the metallic elements that had a greater weight on this PC (Figure 2B). Meanwhile, PC2 (Figure 2A) enabled the separation of the samples of *R. cyanoxantha* and those of the genus *Boletus* amongst them. In this case, Cr and Zn were the metallic elements with the major weight on this PC. Based on the PCA results, the *A. caesarea* samples (Figure 2A) were not grouped and separated from the rest of the species. The results obtained from the PCA agreed with those obtained by HCA, indicating a greater tendency for clustering linked to the mushroom species in terms of accumulation of metallic elements and metalloids determined in this study.

### 3.3. Estimated Daily Intake of Metals

To evaluate the potential human health risks associated with the consumption of the wild edible mushroom species studied (*R. cyanoxantha*, *A. caesarea*, *A. silvicola*, *B. aereus*, and *B. edulis*), the estimated daily intake of metals (EDIM) was calculated for the five toxic elements (Cr, As, Cd, Hg, and Pb), as well as for the three essential elements (Cu, Zn, and Se). For this purpose, a 300-g portion of fresh mushrooms (30 g of dried mushrooms) and an average consumer of 70 kg were assumed. The EDIM results obtained (Table 4) were compared with the values established by the Joint FAO/WHO Expert Committee on Food Additives (JECFA) for the Provisional Tolerable Maximum Daily Intake (PTMDI) and the Provisional Tolerable Daily Intake (PTDI) for Cu, Zn, As, Cd, and Hg, and with the R_f_D values established for Se, Cr, and Pb.

On a global basis, the highest EDIM values observed were for Zn, and specifically, for sample #8 *A. silvicola* collected in Parc Naturel Bouhachem (Chaouen, Morocco; 91.4 μg kg body weight^−1^ per day). Nevertheless, when comparing the EDIMs obtained for this metallic element with the PTMDI (300–1000 μg kg body weight^−1^ per day), it was noted that all were found to be below the established value. Similarly, it was observed that the EDIMs for Cu were generally below the established PTMDI (5000 μg kg body weight^−1^ per day). On the other hand, the EDIMs values obtained for Se were found to be generally above the R_f_D established for this metalloid (0.5 μg kg body weight^−1^ per day), except for samples #*1 R. cyanoxantha* from Cortes de la Frontera (Malaga, Spain; 0.263 μg kg body weight^−1^ per day), #7 *A. caesarea* from Cortes de la Frontera (Malaga, Spain; 0. 410 μg kg body weight^−1^ per day), #8 *A. silvicola* from Parc Naturel Bouhachem (Chaouen, Morocco; 0.322 μg kg body weight^−1^ per day), #13 *B. aereus* from Valdeinfierno (Cadiz, Spain; 0.119 μg kg body weight^−1^ per day), and #15 *B. aereus* from Parc Naturel Bouhachem (Chaouen, Morocco; 0.485 μg kg body weight^−1^ per day). Regarding Cr, the EDIMs obtained were mainly under R_f_D (3 μg kg body weight^−1^ per day), except for samples #*1 R. cyanoxantha* from Cortes de la Frontera (Malaga, Spain; 3.17 μg kg body weight^−1^ per day), #3 *R. cyanoxantha* from Cortes de la Frontera (Malaga, Spain; 4.40 μg kg body weight^−1^ per day), and #7 *A. caesarea* from Cortes de la Frontera (Malaga, Spain; 4.29 μg kg body weight^−1^ per day). From the results obtained through this study, it is worth noting that the highest EDIMs for Cr have been detected in all cases in samples collected in Cortes de la Frontera (Malaga, Spain). The EDIMs obtained for As were found to be below the established PTDI (2.14 μg kg body weight^−1^ per day) for all the samples studied. Likewise, EDIMs for Pb were also under the established R_f_D value (3.5 μg kg body weight^−1^ per day) for all mushroom species analyzed in this study. Additionally, the EDIMs obtained for Cd were found to be below the established PTDI (0.82 μg kg body weight^−1^ per day), except for samples #4 *A. caesarea* from Puerto de Galiz (Cadiz, Spain; 1.73 μg kg body weight^−1^ per day), #5 *A. cesarea* from Puerto de Galiz (Cadiz, Spain; 1.20 μg kg body weight^−1^ per day), #6 *A. caesarea* from Sendero El Palancar (Cadiz, Spain; 0. 895 μg kg body weight^−1^ per day), #8 *A. silvicola* from Parc Naturel Bouhachem (Chaouen, Morocco; 5.79 μg kg body weight^−1^ per day), #9 *A. silvicola* (11.1 μg kg body weight^−1^ per day), and #10 *A. silvicola* (8.89 μg kg body weight^−1^ per day) both from Cortes de la Frontera (Malaga, Spain). In contrast, the EDIMS obtained for Hg were found to be above the PTDI (0.57 μg kg body weight^−1^ per day), apart from sample #8 *A. silvicola* from Parc Naturel Bouhachem (Chaouen, Morocco; 0.512 μg kg body weight^−1^ per day). 

### 3.4. Health Risk Assessment

**Figure 3:**
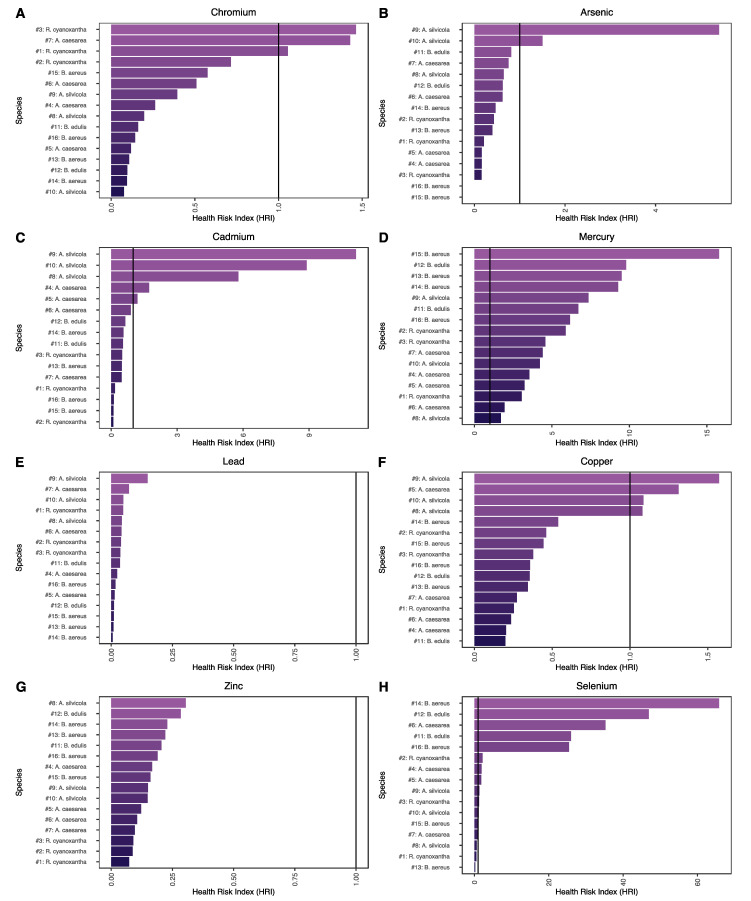
Bar charts of the Health Risk Index (HRI) result according to metallic elements and metalloids determined for all the wild edible mushroom species studied: (**A**) Cr HRIs; (**B**) As HRIs; (**C**) Cd HRIs; (**D**) Hg HRIs; (**E**) Pb HRIs; (**F**) Cu HRIs; (**G**) Zn HRIs; (**H**) Se HRIs. The HRI limit has been represented with the help of a vertical black line in the bar charts.

The assessment of the potential health risk was performed using the Health Risk Index (HRI) calculation, which is the ratio between the EDIM for each of the samples studied and the R_f_D for each element. The results have been graphically displayed in bar charts as shown in Figure 3. HRI values equal to or less than 1 for a given element would indicate that the consumption of a particular mushroom species collected from a given geographic location is considered safe for the consumer [2,14]. This limit has been represented with the help of a vertical black line in the bar charts. According to the results shown in Figure 3, it should be highlighted that only Zn and Pb presented HRIs ≤ 1 for all the samples analyzed, indicating that the consumption of the studied mushrooms from these sample sites would be exempt from health risks, and especially for Pb, which is a probable human carcinogen, Group 2A, according to the International Agency for Research on Cancer (IARC) [47]. Regarding the remaining metallic elements and metalloids, the HRIs were found to be above the criteria for safe consumption (HRI ≤ 1) established for all or part of the mushrooms studied. Specifically, the highest HRI values in this study were observed for Se and, secondly, for Hg. For both cases, the calculated HRIs were considerably above the established safe consumption criterion. In general, the HRIs calculated for Se (Figure 3H) were found to be above 1, except for samples #1 *R. cyanoxantha* from Cortes de la Frontera (Malaga, Spain), #7 *A. caesarea* from Cortes de la Frontera (Malaga, Spain), #8 *A. silvicola* from Parc Naturel Bouhachem (Chaouen, Morocco), #13 *B. aereus* from Valdeinfierno (Cadiz, Spain), and #15 *B. aereus* from Parc Naturel Bouhachem (Chaouen, Morocco), of which the HRI values were found to be below 1. Se is a metalloid classified as an essential trace element, necessary for the organism’s normal functioning. Selenium intoxication due to an overdose is generally rare, especially if it comes from food sources. However, selenium intake above the recommended dose may contribute to the prevention of prostate cancer [48,49]. Therefore, there is no evidence that the consumption of the mushroom species from the geographical areas studied with HRIs greater than 1 may pose a health threat related to this metalloid. 

Regarding Hg, all the samples studied showed HRI values higher than 1 (Figure 3D). Hg is a highly toxic metallic element that is present in mercury-based organic compounds, including methylmercury, and in many inorganic forms, such as metallic mercury (Hg^0^) and mercurous (Hg^2++^) or mercuric (Hg^++^) salts. The main organs that are potentially affected by mercury and mercurial salts are the intestinal lining and the kidneys, whereas methylmercury is widely distributed throughout the organism [50]. Nowadays, metallic Hg and inorganic Hg compounds are classified by IARC in Group 3 as not classifiable as to their carcinogenicity to humans and methylmercury compounds as possibly carcinogenic to humans [51]. Since no chemical speciation was carried out in this work and due to the toxicity of the different forms in which Hg occurs, the consumption of the mushrooms studied from the geographic locations sampled may represent a risk to human health in terms of exposure to this metallic element during the mushrooming season. On the other hand, the HRIs calculated for Cu (Figure 3F) were mostly below 1, except for samples #5 *A. caesarea* from Puerto de Galiz (Cadiz, Spain); #8 *A. silvicola* from Parc Naturel Bouhachem (Chaouen, Morocco); and #9 and #10, which were both *A. silvicola* from Cortes de la Frontera (Malaga, Spain), which all had HRIs was above 1. Cu is a metallic element classified as an essential trace element for humans occurring in many enzymes which are important in various systems, such as the immune and nervous systems. Nonetheless, it may still pose somewhat of a risk to human health at elevated levels of exposure, mainly in the gastrointestinal tract [52]. According to the results obtained in this research, it cannot be excluded that prolonged exposure to this metallic element through consumption of the mushrooms with an HRI > 1 may have health repercussions for the consumers. Nevertheless, moderate consumption throughout the mushrooming season should not necessarily have adverse health implications associated with this essential metallic element, since it is necessary for the proper functioning of the organism at safe concentrations. 

The HRI values for Cr (Figure 3A) were observed below 1, except in samples #1 and #3 of *R. cyanoxantha* and # 7 of *A. caesarea*, with all of them collected in Cortes de la Frontera (Malaga, Spain). Cr is a metallic element that can occur in two forms: trivalent chromium (Cr^3+^) and hexavalent chromium (Cr^6+^). The trivalent form is considered an essential element for the proper functioning of the organism, since it participates in the metabolism of glucose, cholesterol, and fatty acids. However, its hexavalent form is highly reactive, constituting a form of toxicological concern due to its adverse effects on the body, such as disorders in the respiratory and digestive systems [53,54]. Consequently, Cr^3+^ has been classified by the IARC in Group 3 as unclassifiable regarding its carcinogenicity to humans and Cr^6+^ in Group 1 as potentially carcinogenic to humans [53]. In the present study, Cr was determined as the total Cr. Notwithstanding, due to toxicological concerns associated with one of the forms of this metallic element and considering that the mutual transitions from one form to the other are quite dynamic, it is not possible to exclude health risks associated with Cr exposure through the consumption of mushrooms with HRIs > 1. For As, the HRIs values (Figure 3B) were found to be below 1, except for samples #9 and #10 *A. silvicola,* which were both collected in Cortes de la Frontera (Malaga, Spain). Arsenic is a metalloid element causing adverse health effects due to its high toxicity in its inorganic form, specifically in its trivalent (As^3+^) and pentavalent (As^5+^) states. Inorganic arsenic has been classified by the Agency for Toxic Substances and Disease Registry (ASTDR) as a Group 1 carcinogen for humans [55]. Therefore, the consumption of such mushrooms that present an HRI < 1 may pose a risk to human health in terms of exposure to this metalloid. Regarding Cd (Figure 3C), the HRIs calculated were below 1, except for samples #4 and #5 *A. caesarea* from Puerto de Galiz (Cadiz, Spain), #8 *A. silvicola* from Parc Naturel Bouhachem (Chaouen, Morocco), and #9 and #10 *A. silvicola* from Cortes de la Frontera (Malaga, Spain). Cd is a metallic element that is mainly found in its bivalent state (Cd^2+^) and accumulates in tissues and organs and can cause serious diseases, such as cancer [56]. Thus, Cd and its compounds have been classified as Group 1 carcinogenic to humans by the IARC [57]. Due to the toxicological attention concerning Cd, the consumption of those samples with HRI > 1 may pose a health risk in terms of this metallic element.

## 4. Conclusions

The total content of five toxic elements (Cr, As, Cd, Hg, and Pb) and three essential elements (Cu, Zn, and Se) was determined in wild-growing edible mushrooms (*A. silvicola, A. caesarea, B. aereus, B. edulis, and R. cyanoxantha*) collected in southern Spain and northern Morocco. Among the metallic elements and metalloids determined, Zn was the most abundant, followed by Cu and Se. The rest of the elements were found in relatively lower concentrations. The application of multivariate techniques, such as Hierarchical Cluster Analysis (HCA) and Principal Component Analysis (PCA), indicated that there are considerable differences in the uptake of metallic elements and metalloids related to species-intrinsic factors. On the other hand, consumer exposure and health implications were assessed using two well-known safety criteria: the Estimated Daily Intake of Metals (EDIM) and the Health Risk Index (HRI). The highest EDIM values obtained were observed for Zn. The HRI assessment for all mushroom species studied showed a concern related to Hg due to the frequent consumption of between 300 g of fresh mushrooms per day. Meanwhile, mushroom species that presented HRIs > 1 for Cr, As, and Cd also pose a cause for concern in terms of exposure to these metallic elements and metalloids. 

As is well recognized, mushroom consumers normally assume that the intake of edible wild mushrooms is risk-free. However, the findings reported in the present study indicate that excessive consumption of the mushroom species studied during the mushrooming season could have health implications and that mushrooms should therefore be consumed moderately.

## Figures and Tables

**Table 1:** Sample ID, mushroom species, family, sample location, collection date, and habitat description of wild edible mushroom species studied.

Sample ID	Mushroom Species	Family	Sample Location	Date	Habitat
#1	*Russula cyanoxantha*	*Russulaceae*	Cortes de la Fra. (Malaga, Spain)	2017	Deciduous forest; *Quercus*
#2			Sendero El Palancar (Cadiz, Spain)	2018	
#3			Cortes de la Fra. (Malaga, Spain)	2018	
#4	*Amanita caesarea*	*Amanitaceae*	Puerto de Galiz (Cadiz, Spain)	2017	Deciduous forest; *Quercus suber*
#5			Puerto de Galiz (Cadiz, Spain)	2018	
#6			Sendero El Palancar (Cadiz, Spain)	2018	
#7			Cortes de la Fra. (Malaga, Spain)	2018	
#8	*Agaricus silvicola*	*Agaricaceae*	Parc Naturel Bouhachem(Chaouen, Morocco)	2017	Deciduous forest; *Quercus suber*
#9			Cortes de la Fra. (Malaga, Spain) ^1^	2018	
#10			Cortes de la Fra. (Malaga, Spain) ^2^	2018	
#11	*Boletus edulis*	*Boletaceae*	Puerto de Galiz (Cadiz, Spain)	2018	Deciduous forest; *Quercus suber*
#12			Valdeinfierno (Cadiz, Spain)	2018	
#13	*Boletus aereus*	*Boleataceae*	Valdeinfierno (Cadiz, Spain)	2018	Deciduous forest; *Quercus suber*
#14			Puerto de Galiz (Cadiz, Spain)	2018	
#15			Parc Naturel Bouhachem(Chaouen, Morocco) ^1^	2017	
#16			Parc Naturel Bouhachem(Chaouen, Morocco) ^2^	2017	

**Table 2:** ICP-MS instrumental conditions for the metallic elements and metalloid determination in the mushroom samples.

ICP-MS Instrumental Conditions	
CCT H2(7%)/He (mL min^–1^)	4.5
Pole Bias Voltage (V)	–17.0
Hexapole Bias Voltage (V)	–20.0
Auxiliary Ar Flow Rate (L min^–1^)	1.0
Nebulizer Ar Flow Rate (L min^–1^)	1.0
Plasma Ar Flow Rate (L min^–1^)	14.0
Sampling depth (mm)	80.0
RF Power	1380 W

**Table 3:** Metallic elements and metalloid concentrations (mg kg^−1^ DW) in the analyzed wild edible mushroom species from southern Spain and northern Morocco.

Sample ID	Cu	Zn	Se	Cr	As	Cd	Hg	Pb
#1	23.7 ± 0.035	51.2 ± 0.873	0.613 ± 0.876	7.39 ± 0.951	0.147 ± 0.008	0.408 ± 0.027	2.14 ± 0.024	0.397 ± 0.032
#2	43.1 ± 2.81	61.4 ± 0.972	2.55 ± 0.102	5.01 ± 0.119	0.302 ± 0.004	0.223 ± 0.030	4.12 ± 0.069	0.325 ± 0.007
#3	35.3 ± 0.254	63.4 ± 1.15	1.49 ± 0.059	10.2 ± 0.079	0.111 ± 0.003	1.16 ± 0.045	3.21 ± 0.091	0.305 ± 0.017
#4	18.9 ± 0.231	117 ± 2.03	2.28 ± 0.002	1.84 ± 0.093	0.113 ± 0.009	4.03 ± 0.006	2.49 ± 0.002	0.199 ± 0.009
#5	123 ± 1.47	85.6 ± 0.624	2.13 ± 0.004	0.828 ± 0.011	0.113 ± 0.009	2.79 ± 0.023	2.27 ± 0.038	0.115 ± 0.013
#6	22.0 ± 0.500	74.1 ± 0.155	41.1 ± 0.761	3.57 ± 0.181	0.436 ± 0.010	2.09 ± 0.039	1.37 ± 0.018	0.342 ± 0.013
#7	25.5 ± 0.512	67.6 ± 1.24	0.958 ± 0.037	10.0 ± 0.026	0.529 ± 0.040	1.10 ± 0.013	3.09 ± 0.064	0.591 ± 0.020
#8	101 ± 1.63	213 ± 3.62	0.750 ± 0.029	1.38 ± 0.010	0.453 ± 0.048	13.5 ± 0.100	1.19 ± 0.023	0.354 ± 0.004
#9	147 ± 2.07	105 ± 1.81	1.57 ± 0.002	2.77 ± 0.991	3.78 ± 0.005	25.9 ± 0.418	5.16 ± 0.153	1.21 ± 0.019
#10	102 ± 5.90	104 ± 5.95	1.38 ± 0.049	0.531 ± 0.051	1.05 ± 0.046	20.8 ± 1.39	2.96 ± 0.175	0.405 ± 0.030
#11	18.6 ± 0.257	144 ± 2.21	30.3 ± 0.686	1.13 ± 0.308	0.570 ± 0.002	1.25 ± 0.034	4.70 ± 0.126	0.293 ± 0.013
#12	33.2 ± 0.034	199 ± 0.245	54.7 ± 0.201	0.675 ± 0.032	0.439 ± 0.002	1.50 ± 0.035	6.85 ± 0.147	0.094 ± 0.008
#13	32.1 ± 0.181	155 ± 1.34	0.278 ± 0.003	0.750 ± 0.023	0.278 ± 0.003	1.13 ± 0.001	6.66 ± 0.073	0.078 ± 0.001
#14	50.3 ± 0.737	160 ± 1.78	76.8 ± 1.46	0.660 ± 0.007	0.328 ± 0.007	1.30 ± 0.021	6.49 ± 0.050	0.047 ± 0.003
#15	41.4 ± 0.509	112 ± 5.69	1.13 ± 0.154	4.03 ± 0.010	<0.200	0.248 ± 0.007	11.1 ± 0.489	0.090 ± 0.003
#16	33.4 ± 0.065	133 ± 0.729	29.7 ± 0.774	1.00 ± 0.020	<0.200	0.272 ± 0.037	4.32 ± 0.007	0.144 ± 0.002

**Table 4:** Estimated Daily Intake of Metals (EDIM) expressed as μg kg body weight^−1^ per day for the analyzed wild edible mushrooms species from southern Spain and northern Morocco.

Sample ID	Cu	Zn	Se	Cr	As	Cd	Hg	Pb
#1	10.2	21.9	0.263	3.17	0.0629	0.175	0.918	0.170
#2	18.5	26.3	1.09	2.15	0.129	0.0957	1.76	0.139
#3	15.1	27.2	0.640	4.40	0.0475	0.496	1.38	0.131
#4	8.12	50.1	0.976	0.787	0.0483	1.73	1.07	0.085
#5	52.5	36.7	0.912	0.355	0.0485	1.20	0.971	0.049
#6	9.45	31.7	17.62	1.53	0.187	0.895	0.586	0.146
#7	10.9	29.0	0.410	4.29	0.227	0.472	1.32	0.253
#8	43.2	91.4	0.322	0.59	0.194	5.79	0.512	0.152
#9	62.9	45.0	0.671	1.19	1.62	11.1	2.21	0.520
#10	43.5	44.6	0.590	0.228	0.451	8.89	1.27	0.173
#11	7.96	61.6	13.0	0.483	0.244	0.535	2.01	0.126
#12	14.2	85.3	23.5	0.289	0.188	0.644	2.94	0.0401
#13	13.8	66.3	0.119	0.322	0.119	0.486	2.85	0.0332
#14	21.6	68.8	32.9	0.283	0.141	0.556	2.78	0.0203
#15	17.8	48.0	0.485	1.73	n.d.	0.106	4.74	0.0385
#16	14.3	56.9	12.7	0.429	n.d.	0.117	1.85	0.0618
RfD ^a^ (μg kg body weight-1 per day)	40 ^d^	300 ^e^	0.5 ^e^	3 ^d^	0.3 ^d^	1 ^d^	0.3 ^d^	3.5 ^e^
PTDI ^b^ (μg kg body weight-1 per day)	-	-	-	-	2.14 ^f^	0.82 ^f^	0.57 ^f^	-
PTMDI ^c^ (μg kg body weight^−1^ per day)	5000 ^f^	300–1000 ^f^	-	-	-	-	-	-

^a^ R_f_D: Reference dose. ^b^ PTDI: Provisional tolerable daily intake. ^c^ PTMDI: provisional maximum tolerable daily intake. ^d^ Sarikurkcu, C. et al. (2020) [2]. ^e^ USEPA: U.S. Environmental Protection Agency [45]. ^f^ JECEFA:The Joint FAO/WHO Expert Committee on Food Additives [46].

## Data Availability

Please refer to suggested Data Availability Statements in section “MDPI Research Data Policies” at https://www.mdpi.com/ethics (accessed on 22 April 2022).
